# Cost-effectiveness analysis of once-daily oral semaglutide versus placebo and subcutaneous glucagon-like peptide-1 receptor agonists added to insulin in patients with type 2 diabetes in China

**DOI:** 10.3389/fphar.2023.1226778

**Published:** 2023-08-09

**Authors:** Zhen Feng, Wai Kei Tong, Xinyue Zhang, Zhijia Tang

**Affiliations:** ^1^ Department of Clinical Pharmacy and Pharmacy Administration, School of Pharmacy, Fudan University, Shanghai, China; ^2^ Department of Pharmacy, The Affiliated Hospital of Xuzhou Medical University, Xuzhou, Jiangsu, China

**Keywords:** cost-effectiveness, semaglutide, GLP-1 receptor agonists, type 2 diabetes, China

## Abstract

**Introduction:** Oral semaglutide is a glucagon-like peptide-1 receptor agonist (GLP-1 RA) that improves glycated hemoglobin levels and body weight in patients with type 2 diabetes (T2DM). We aim to evaluate the cost-effectiveness of once-daily oral semaglutide in comparison to placebo and injectable GLP-1 RAs in Chinese patients with T2DM inadequately controlled on basal insulin.

**Methods:** The United Kingdom Prospective Diabetes Study Outcomes Model (UKPDS OM2.1) was used to estimate the cost-effectiveness by calculating the incremental cost-effectiveness ratio (ICER). Baseline characteristics of the simulation cohort were obtained from the PIONEER 8 trial. Utility and safety inputs were derived from a network meta-analysis of 12 trials. Direct medical costs were retrieved from published literature and discounted at an annual rate of 5%. We used a willingness-to-pay (WTP) threshold of $36,528.3 per quality-adjusted life-year (QALY) gained. Scenario analysis, and one-way and probabilistic sensitivity analysis were performed.

**Results:** The effectiveness of oral semaglutide was 10.39 QALYs with a total cost of $30,223.10, while placebo provided 10.13 QALYs at a lower total cost of $20,039.19. Oral semaglutide was not cost-effective at an ICER of $39,853.22 and $88,776.61 per QALY compared to placebo and exenatide at the WTP. However, at an annual price of $1,871.9, it was cost-effective compared with dulaglutide, liraglutide, and lixisenatide. The model was most sensitive to the discount rate and annual cost of oral semaglutide. The price of oral semaglutide needed to be reduced to $1,711.03 per year to be cost-effective compared to placebo and other injectable GLP-1 RAs except for exenatide and semaglutide injection.

**Conclusion:** We found that once-daily oral semaglutide, at a comparable price of semaglutide injection, proves to be a cost-effective add-on therapy to insulin for Chinese patients with T2DM, especially when compared to subcutaneous GLP-1 RAs other than injectable semaglutide and exenatide. However, to achieve cost-effectiveness in comparison to placebo, further cost reduction of oral semaglutide is necessary. The estimated annual cost of $1,711.03 for oral semaglutide demonstrates a more cost-effective option than placebo, highlighting its potential value in the management of T2DM.

## 1 Introduction

Globally, diabetes has emerged as a pressing public health concern, affecting approximately 537 million individuals worldwide, predominantly with type 2 diabetes mellitus (T2DM) (Ahmad et al., 2022). China has the highest number of diabetes patients in the world, and its prevalence has surged considerably in recent decades. The economic burden of diabetes on healthcare budgets is substantial and projected to remain so in the future (Wang et al., 2018). It is estimated that total diabetes-related healthcare expenditure in China has escalated from $51 billion in $2015 to $109 billion in 2019 (Zhang, 2022). This increase in cost can be partially attributed to the utilization of novel classes of antidiabetic medications, such as glucagon-like peptide-1 receptor agonists (GLP-1 RAs) (Choi et al., 2022).

The latest guidelines propose new recommendations for the management of T2DM, comprising patient education for adopting healthier lifestyles (e.g., physical activity and caloric restriction) and improving glycemic control (American Diabetes Association Professional Practice Committee, 2022; Davis et al., 2022). Nonetheless, achieving optimal glycemic targets remains challenging for some patients, despite increasing insulin doses, due to unfavorable effects on body weight and adverse reactions (Swinnen et al., 2009; Rodbard et al., 2018). Additionally, insulin exerts little impact on cardiovascular complications, a key contributor to morbidity and mortality in patients with diabetes (Zheng et al., 2018).

GLP-1 RAs have demonstrated clinical efficacy as first-line therapy for reducing HbA1c and body weight in T2DM patients who are at high risk for cardiovascular disease. These benefits have been observed when GLP-1 RAs are used as add-on therapy to oral hypoglycemic agents or in combination with basal insulin, as evidenced by several recent studies (Artigas et al., 2015; Bucheit et al., 2020; Nauck et al., 2021; ElSayed et al., 2023). Additionally, GLP-1 RAs have demonstrated cardiovascular safety profile in individuals with diabetes (Marso et al., 2016; Husain et al., 2019; Kristensen et al., 2019; Bucheit et al., 2020; Ghosh-Swaby et al., 2020; Ma et al., 2021). However, their utilization is hindered by the high costs compared to insulin and other oral agents, leading to concerns regarding the trade-off between clinical benefits and expenses, especially in regions like Taiwan, where the cost per treatment cycle may be up to 7 times that of insulin (Yang et al., 2020). Moreover, the inconvenience and discomfort associated with subcutaneous injection could negatively impact medication adherence (Bucheit et al., 2020). Despite numerous studies comparing different GLP-1 RAs, variances in inclusion criteria (e.g., baseline HbA1c and background medications), study duration, and analysis methods have rendered it challenging to establish meaningful comparisons between individual trials.

Oral formulations of GLP-1 RAs enhance treatment convenience, acceptance, and adherence, offering patients an alternative option to achieve glycemic goals without injections (Meier, 2021). In September 2019, oral semaglutide, the world’s first and only GLP-1 RA with both injection and oral dosage forms, was approved by the FDA for treating T2DM. The cardiovascular safety of oral semaglutide has been established in patients at high risk of cardiovascular disease or with existing cardiovascular disease (Husain et al., 2019; Thethi et al., 2020). Oral semaglutide has similar efficacy and safety profiles to the injectable formulation, with no additional cardiovascular risk compared to placebo (Davies et al., 2017; Husain et al., 2019), and neither formulation increases the risk of hypoglycemia while improving the quality of life (Meier, 2021). Although China’s National Medical Products Administration (NMPA) has yet to approve oral semaglutide, the injection formulation has been included in China’s national medical insurance since 2021, reimbursing patients 90% of the drug price. Given diabetes’s adverse outcomes and economic burden, Chinese healthcare policymakers must weigh the clinical benefits of GLP-1 RAs against their high costs.

Currently, there is a lack of data regarding the cost-effectiveness and long-term diabetes-related outcomes of oral semaglutide compared to other subcutaneous GLP-1 RAs. This study aimed to evaluate the cost-effectiveness and long-term outcomes of oral semaglutide versus placebo and other injectable GLP-1 RAs listed in China’s national medical insurance catalogue, including semaglutide, dulaglutide, exenatide, liraglutide, and lixisenatide. We hypothesized that the oral formulation of semaglutide would be non-inferior to injectable formulations. Additionally, this study aimed to determine the appropriate pricing of once-daily oral semaglutide in China.

## 2 Materials and methods

### 2.1 Model overview

The United Kingdom Prospective Diabetes Study Outcome Model version 2.1 (UKPDS OM2) is a validated computerized simulation tool for evaluating the long-term effects of interventions and complications in patients with T2DM, as well as economic outcomes (DTU, 2019). It has been applied globally (Clarke et al., 2005; Cunningham et al., 2022), including the Chinese T2DM population (Hu et al., 2021; Hu et al., 2022a). The model is based on data from 5,102 participants in the 20-year trial, and 4,031 survivors with a 10-year post-trial monitoring (PTM) follow-up period, which is used to model the risk factor progression equation (Hayes et al., 2013). Model inputs include demographic characteristics (i.e., age, gender, race, diabetes duration, weight, and height), risk factors (e.g., HbA1c, heart rate, hemoglobin, and white blood count), pre-existing events (e.g., amputation, blindness, and renal failure), cost and utility parameters. Model outputs include estimated life years (LY), quality-adjusted life years (QALY), therapy costs, complication costs, and total costs. The model’s structure and algorithm have been previously described (Hayes et al., 2013).

### 2.2 Simulation population and treatment inputs

We utilized patient characteristics and treatments from a network meta-analysis comparing once-daily oral semaglutide 14 mg to injectable GLP-1 RAs in individuals with poorly controlled T2DM on basal insulin. The meta-analysis evaluated the relative efficacy and safety of various GLP-1 RAs, including dulaglutide, exenatide, liraglutide, lixisenatide, and once-weekly semaglutide injections (Chubb et al., 2021). It is not in line with either previously published data or real-world practice to maintain GLP-1 receptor agonist therapy for patients’ lifetimes (Divino et al., 2017; Malkin et al., 2022). Therefore, we assumed that patients would be treated for 5 years, which is much shorter than the lifetime assumptions. The duration of GLP-1 RA treatment in the present study was similar to that of previous reports (Hu et al., 2021; Hu et al., 2022a; Hu et al., 2022b).

The model parameters were derived from data obtained from the PIONEER 8 trial and additional unreported data from UKPDS (Hayes et al., 2013). The environment of the above meta-analysis was comparable to that of the PIONEER 8 trial, which allowed for generalization to the target population of this study. Supplementary Table S1 displays the baseline characteristics of a simulated cohort comprising 1,000 subjects in each intervention group, with a mean age of 61.0 ± 10.0 years and a mean HbA1c of 8.2% ± 0.7%. Supplementary Table S2 illustrates the treatment differences between oral semaglutide and comparator drugs in the network meta-analysis. The intervention involved a once-daily administration of oral semaglutide at dose of 14 mg, while the comparators consisted of once-weekly injectable semaglutide at 1.0 mg, once-weekly dulaglutide at 1.5 mg, once-daily liraglutide at 1.8 mg, twice-daily exenatide at 10 ug, and once-daily lixisenatide at 20 ug (Supplementary Table S2).

### 2.3 Cost and utilities

This study was conducted from the perspective of Chinese healthcare payers; therefore, only direct medical costs associated with T2DM and its complications were calculated. Drug procurement costs were based on 2022 public hospital procurement prices (Supplementary Table S3). Despite oral semaglutide being unavailable in mainland China, given that the pack prices of oral semaglutide 14 mg and injectable semaglutide 1 mg are the same in the United States (Hansen et al., 2020), this study still considered the prices of the two dosage forms to be equal. Costs related to diabetes complications and management, as well as health status, were derived from a Chinese diabetes population assessment (Supplementary Table S4) (Hu et al., 2022a; Hu et al., 2022b). The analysis excluded costs for basal insulin, background oral medications, adverse event management, and out-of-pocket injection fees, which were assumed to be consistent across groups. Other unknown data were derived from default values provided in the UKPDS OM2 and UKPDS 62 studies (Clarke et al., 2002). The initial utility value for T2DM patients was 0.876 (Pan et al., 2016). All amounts were expressed in Chinese yuan (CNY) and converted to U.S. dollars (USD) using the average exchange rate from January to October 2022 (1 USD = 6.6504 CNY).

### 2.4 Discounting and time horizon

Discounting was applied to both costs and outcomes at an annual rate of 5%. The time horizon for base case analysis was set at 40 years, which corresponded to the mean age of the simulated cohort and was deemed sufficient to capture lifetime cost-effectiveness.

### 2.5 Pharmacoeconomic assessment

The incremental cost-effectiveness ratio (ICER) was calculated from the model output by dividing the incremental cost by the incremental QALY. To assess cost-effectiveness, a willingness-to-pay (WTP) threshold of three times the gross domestic product (GDP) *per capita* was used, which amounted to $36,528.3 per QALY gained in this study, following the recommendations of the World Health Organization (WHO) (Hutubessy et al., 2003; Marseille et al., 2015; Bertram et al., 2016).

### 2.6 Sensitivity analysis and price threshold analysis

We performed a univariate sensitivity analysis to evaluate the impact of changing key inputs in the model, including the discount rate, initial utility, time horizon, costs, and disutility scores (Supplementary Table S5). Tornado diagrams were created to visualize the impact of these changes in oral semaglutide compared to placebo, with 95% confidence intervals (CIs) of the corresponding estimates and ±20% of the base case values as upper and lower bounds (Shafie and Ng, 2020; Hu et al., 2022a). To estimate the cost-effectiveness acceptability curve (CEAC), we employed a probabilistic sensitivity analysis (PSA) using the Monte Carlo method. This involved conducting 1,000 simulations, with input parameters sampled from a fixed probability distribution to address second-order uncertainty. Additionally, we conducted a scenario analysis to evaluate the effect of varying simulation duration, and a threshold analysis to suggest an appropriate price for oral semaglutide to enter the Chinese market. We employed a binary search approach to determine the appropriate price reduction for oral semaglutide that would result in the ICER of oral semaglutide versus placebo falling below the predetermined WTP threshold (Hu et al., 2021). More specifically, the binary search algorithm began by evaluating the midpoint of the sequence for a decrease in price. By comparing this midpoint value to the target value, we determined if the ICER was higher or lower than the target. This approach effectively halved the search space. We then repeated this iterative process until we achieved the desired price reduction, which equated the ICER of oral semaglutide versus placebo with the WTP threshold.

Our study adheres to the Consolidated Health Economic Evaluation Reporting Standards (CHEERS) reporting guideline for economic evaluations.

## 3 Results

### 3.1 Base-case analysis

Table 1 summarizes the main findings of a 40-year simulation using the UKPDS model. The life expectancy ranged from 11.97 life years in the placebo group to 12.27 life years in the semaglutide injection group. Accounting for quality of life, patients receiving semaglutide injection achieved the highest QALYs at 10.43, representing an additional 0.04 QALYs compared to the oral formulation. The oral semaglutide arm demonstrated improvements of 0.02, 0.23 and 0.11 QALYs compared to the liraglutide, lixisenatide and dulaglutide arms, respectively. The use of oral semaglutide incurred an additional cost of $742.88, $5,006.73, and $3,706.50, resulting in ICERs of $33,041.06/QALY, $21668.64/QALY, and $34,061.37/QALY, when compared to patients receiving liraglutide, lixisenatide and dulaglutide, respectively. Compared to placebo, oral semaglutide was associated with an additional cost of $10,183.91, resulting in ICERs of $38,675.49/LY and $39,853.22/QALY, which exceeded our preset WTP threshold. Furthermore, the use of oral semaglutide incurred additional cost of $5,975.05, with the highest ICER of $88,776.61/QALY, compared to patients receiving exenatide.

**TABLE 1:** Results of base-case analysis.

Outcomes	Oral SEMA	Sc. SEMA	DULA	LIRA	EXEN	LIXI	Placebo
LY, years	12.23	12.27	12.14	12.21	12.18	11.98	11.97
QALY, years	10.39	10.43	10.28	10.36	10.32	10.15	10.13
Treatment costs	10,027.91	10,010.77	6,286.52	9,257.86	3,998.82	5,202.86	0
Complication costs	20,195.19	20,218.22	20,230.09	20,222.37	20,249.23	20,013.51	20,039.19
Total costs	30,223.10	30,228.99	26,516.60	29,480.23	24,248.06	25,216.37	20,039.19
^a^Difference in total costs	NA	−5.89	3,706.50	742.88	5,975.05	5,006.73	10,183.91
^a^Difference in QALY, years	NA	−0.04	0.11	0.02	0.07	0.23	0.26
ICER, $/years							
Per LY	NA	NA	42,404.04	48,892.17	118,364.51	20,318.66	38,675.49
Per QALY	NA	NA	34,061.37	33,041.06	88,776.61	21,668.64	39,853.22

^a^ Difference between oral semaglutide and comparators. Abbreviations: SEMA, semaglutide; Sc, subcutaneous; DULA, dulaglutide; LIRA, liraglutide; EXEN, exenatide; LIXI, lixisenatide; LY, estimated life years; QALY, quality-adjusted life years; ICER, incremental cost-effectiveness ratio; NA, not applicable.

### 3.2 Sensitivity analysis

**FIGURE 1:**
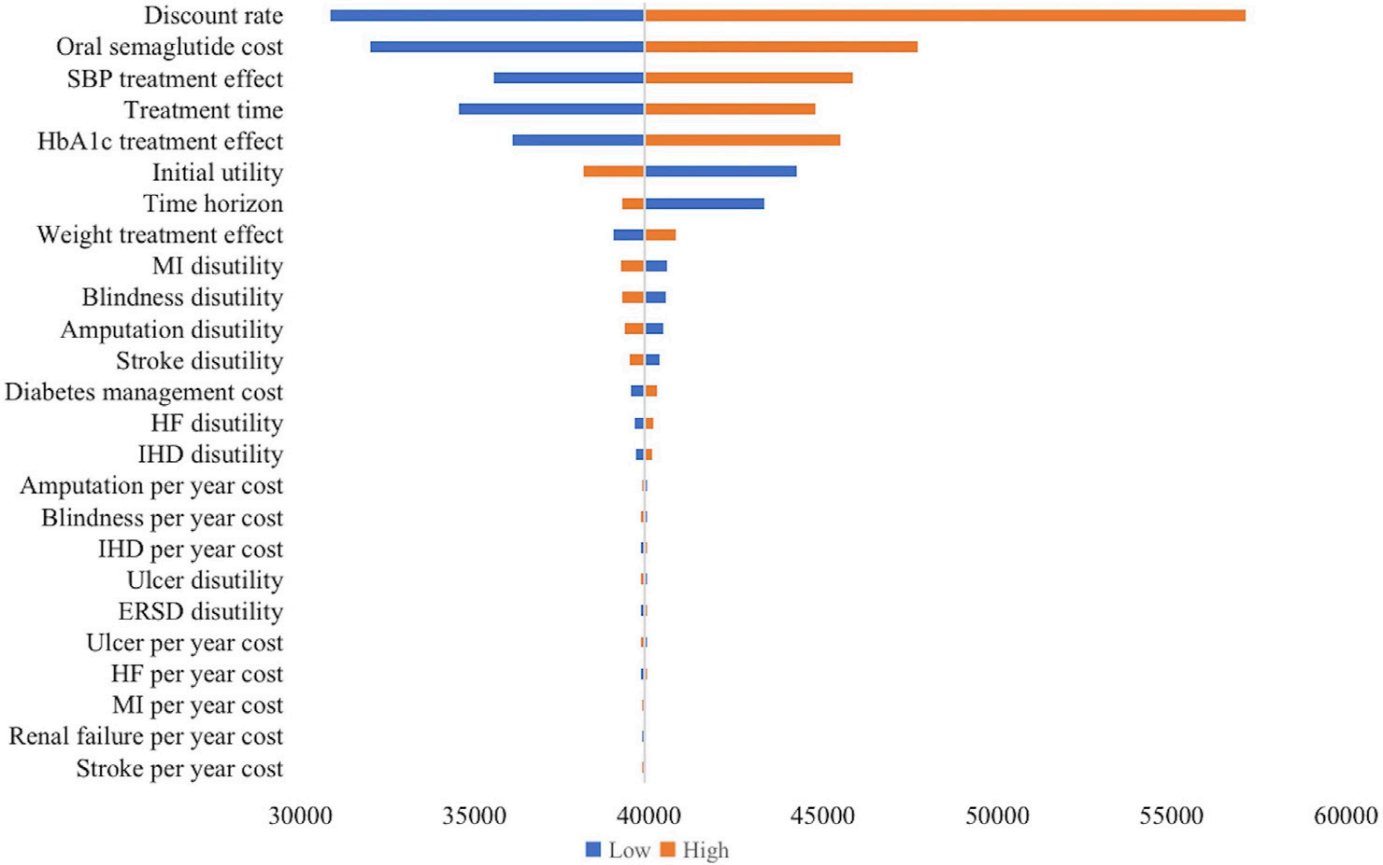
Tornado diagram of one-way sensitivity analysis (oral semaglutide vs. placebo). The influential factors were arranged in descending order of the variation. *X*-axis indicated the ICER. SBP, systolic blood pressure; MI, myocardial infarction; HF, heart failure; IHD, ischemic heart disease; ERSD, End-Stage Renal Disease; ICER, incremental cost-effectiveness ratio.

Figure 1 shows the results of the one-way sensitivity analysis. The variables that had the most significant effect on the ICER were the discount rate (ICER range: $30,857.45-$57,117.71), the annual cost of oral semaglutide (ICER range: $32,004.67-$47,701.77), absolute therapeutic effect on systolic blood pressure (SBP) (ICER range: $35,539.79-$45,856.96), duration of treatment (ICER range: $34,536.96-$44,763.10), and HbA1c treatment effect (ICER range: $36,071.16-$455,01.91). Conversely, other variables, such as disutility scores and complication costs, had moderate to minimal effects on the ICER.

**FIGURE 2:**
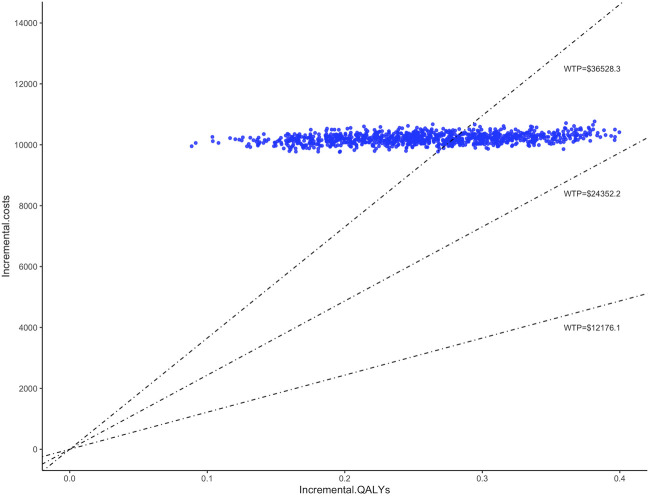
Incremental cost-effectiveness scatter plot. WTP, willingness to pay.

**FIGURE 3:**
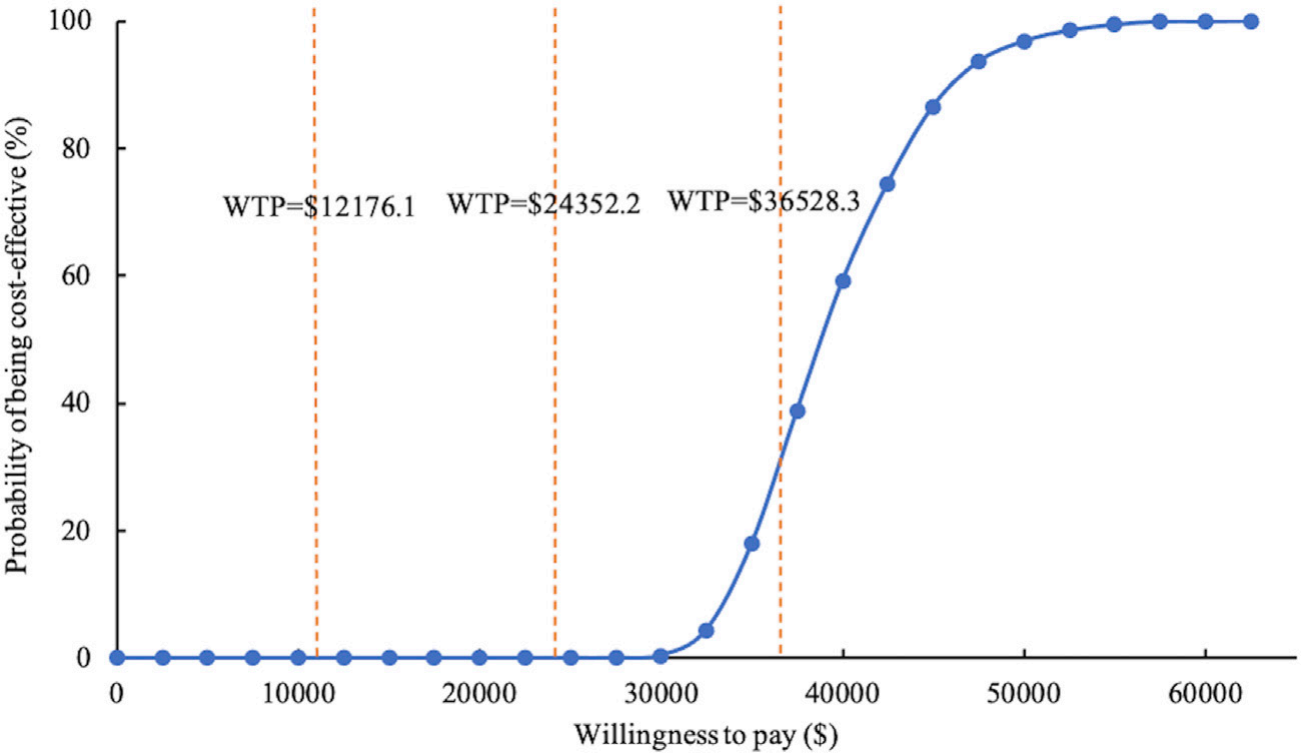
Cost-effectiveness acceptability curve of oral semaglutide vs. placebo. WTP, willingness to pay.

The study conducted 1,000 Monte Carlo simulations and presented the results on an incremental cost-effectiveness scatter plot (Figure 2). To further explore whether oral semaglutide can be cost-effective under different thresholds, we additionally set the WTP to one ($12,176.1 per QALY) to two times ($24,352.2 per QALY) China’s GDP *per capita* in 2022. Cost-effectiveness acceptability curves (Figure 3) were used to assess the probability of oral semaglutide intervention being cost-effective compared to placebo at different thresholds. Results showed that the probability of oral semaglutide being cost-effective was 0% at a WTP of $12,176.1 and $24,352.2 per QALY. At a WTP of $36,528.3/QALY, oral semaglutide had a 17.9% probability of being cost-effective, while at a threshold of $50,000/QALY, the probability increased to 96.8%.

### 3.3 Scenario analysis

In the scenario analysis, we employed five distinct time horizons to assess uncertainties. As demonstrated in Table 2, semaglutide was more cost-effective than all other GLP-1 RAs over a 30-year simulation period. However, for shorter time horizons (<20 years), dulaglutide was more cost-effective. In contrast, irrespective of the simulation time, oral semaglutide was found to be less cost-effective compared to exenatide and placebo. Concerning comparisons between different formulations, the semaglutide injection consistently exhibited greater cost-effectiveness than the oral formulation for simulation periods of less than 30 years. Conversely, the oral formulation exhibited greater cost-effectiveness in the long run.

**TABLE 2:** Results of scenario analysis on time horizon.

ICER, $/QALY	Simulation time horizons				
	10 years	20 years	30 years	40 years	50 years
Oral SEMA vs. Sc. SEMA	Dominated^a^	Dominated^a^	Dominated^a^	NA	NA
Oral SEMA vs. DULA	145,861.22	45,453.14	36,528.25	34,061.37	32,822.89
Oral SEMA vs. LIRA	150,463.68	33,921.43	31,052.70	33,041.06	33,156.71
Oral SEMA vs. EXEN	336,966.85	104,002.37	86,031.63	88,776.61	84,323.97
Oral SEMA vs. LIXI	106,539.38	31,971.99	23,502.68	21,668.64	21,113.42
Oral SEMA vs. Placebo	189,711.78	59,034.44	43,311.98	39,853.22	39,235.55

^a^ In favor of Sc. SEMA. Abbreviations: SEMA, semaglutide; Sc, subcutaneous; DULA, dulaglutide; LIRA, liraglutide; EXEN, exenatide; LIXI, lixisenatide; QALY, quality-adjusted life years; ICER, incremental cost-effectiveness ratio; NA, not applicable.

### 3.4 Price threshold analysis

From the perspective of healthcare payers in China, further reductions in the cost of oral semaglutide are needed to make it a cost-effective therapy compared to placebo. To achieve cost-effectiveness, the market price of oral semaglutide would need to decrease by 8.6% to $1,711.03 per year, resulting in an ICER of $36,480.62/QALY, which falls at the borderline of the preset WTP threshold. At this price point, oral semaglutide becomes a cost-effective option not only when compared to placebo but also to dulaglutide (ICER: $26,141.57/QALY) and lixisenatide (ICER: $17,938.77/QALY). Compared to oral semaglutide at this price, semaglutide injection provided an additional 0.04 QALYs with a higher cost of $867.7, resulting in an ICER of $20,796.80/QALY, which indicates that it remains more cost-effective than oral semaglutide. Additionally, oral semaglutide dominates liraglutide in terms of cost-effectiveness at this price. However, it is still less cost-effective than exenatide (ICER: $75,971.79/QALY).

## 4 Discussion

To our knowledge, this study is the first to evaluate the cost-effectiveness of oral semaglutide 14 mg once daily in Chinese patients with T2DM who are poorly controlled on basal insulin using a diabetes-specific model. The analysis was conducted from the perspective of Chinese payers, and a lifetime horizon was used. Our results demonstrate that oral semaglutide offers good value for money when compared to dulaglutide, liraglutide, and lixisenatide. However, it was inferior to placebo and exenatide when applying a WTP threshold of $36,528.3/QALY. Furthermore, our price analysis revealed that oral semaglutide would be more cost-effective than placebo if the current drug price were to decrease below $1,711.03 per year. In this scenario, treatment costs would be offset by improved quality of life resulting from treatment effects.

While GLP-1 RAs have been found to improve glycemic control and reduce body weight without increasing the risk of hypoglycemia (Buse et al., 2011; Seino et al., 2012; Rodbard et al., 2018), there is limited research on their effects on health-related quality of life. Our study found that all GLP-1 RAs improved life quality when compared to placebo, with semaglutide resulting in the highest LYs and QALYs (Table 1). These findings have clinical implications, especially for patients with T2DM who are concerned about hypoglycemia and weight gain.

As the first and only FDA-approved oral GLP-1 RA product, several studies have assessed the cost-effectiveness of semaglutide for treating T2DM in China, but none have compared its oral formulation with other GLP-1 RAs listed in China’s national medical insurance catalogue (Hu et al., 2021; Hu et al., 2022a; Hu et al., 2022b). Our study shows that over a 40-year period, the cost of semaglutide was higher than other GLP-RAs. For dulaglutide, liraglutide, and lixisenatide, this was more than offset by the incremental utility gained and thus constituted an acceptable ICER. In addition to the previously confirmed cost-effectiveness of injectable semaglutide compared to dulaglutide (Hu et al., 2022a), our study further demonstrated a clear cost-effectiveness of its oral formulation. Studies in the US and Portugal have also confirmed the cost-effectiveness of oral semaglutide compared with dulaglutide (Risebrough et al., 2021; Malkin et al., 2022). Moreover, our model predicted the cost-effectiveness of oral semaglutide compared with liraglutide, with the total incremental QALY (+0.02) consistent with that reported in the literature (0.01–0.07) (Bain et al., 2020; Risebrough et al., 2021). We also estimated that oral semaglutide provided patients with +0.23 QALYs and was more cost-effective than lixisenatide, like a study conducted in Sweden proving that semaglutide injection provided greater clinical benefit (+0.71 QALYs) than lixisenatide in patients with poor basal insulin control at a lower cost (Ericsson and Fridhammar, 2019). These findings were consistent across models, perspectives, and countries, indicating that oral semaglutide is a cost-saving intervention for both policymakers and patients.

Oral semaglutide demonstrated inferior cost-effectiveness compared to exenatide (ICER: $88,776.61/QALY), possibly due to the latter being the first GLP-1 RA available in the Chinese market, with a lower annual price than other GLP-1 RAs (i.e., semaglutide, dulaglutide, liraglutide, and lixisenatide). Although borderline cost-effective against placebo (ICER: $39,853.22/QALY), the overall price of oral semaglutide remains high, underscoring the importance of reasonable drug pricing to improve access to medical resources. In general, when a drug is covered by insurance, its price will drop significantly. Future changes in China’s national medical insurance coverage could prompt a reassessment of these findings.

As a chronic disease, the adherence and persistence of medication regimens are crucial to effectively managing T2DM. Despite their efficacy, GLP-1 RA injections often result in nonadherence due to the inevitable pain and discomfort they induce, leading to patient dissatisfaction (Peyrot et al., 2005; Fu et al., 2009; Kruger et al., 2015; Khunti et al., 2018). Criticisms from patients highlight the need for alternative therapies. While current guidelines in China recommend GLP-1 RAs as second-line therapies, introducing oral GLP-1 analogs would broaden treatment choices for both patients and physicians. The future availability of oral semaglutide in China holds the potential to address the inherent limitations of injections and offer a new, cost-effective treatment option for patients and healthcare payers (Risebrough et al., 2021).

Our study has several limitations. Firstly, we obtained results comparing the efficacy of oral semaglutide and injectable GLP-1 RAs from a network meta-analysis, as direct head-to-head clinical trials were not available. While the methodology aligns with the China Guidelines for Pharmacoeconomic Evaluations (CGPE), further studies involving direct comparisons are still necessary to reassess our findings. Secondly, we aimed to predict long-term outcomes using relatively short-term clinical trial data. Although this approach is common in economic evaluations, caution should be exercised when interpreting the long-term outcomes predicted from relatively short-term clinical trial data. Besides, in the base-case analysis, we assumed a 5-year treatment duration, consistent with other studies (Hu et al., 2021; Hu et al., 2022a; Hu et al., 2022b). However, in real-world situations, patients may adjust their regimens based on blood glucose levels. Our findings were also sensitive to treatment duration with the ICER varying below or above the threshold in sensitivity analysis (oral semaglutide vs. placebo, Figure 1). Thirdly, it is essential to acknowledge that although our study utilized the validated UKPDS-OM2 model for analysis, improvements in HbA1c, body weight, and SBP resulting from diabetes medications may not necessarily guarantee positive long-term outcomes. Future studies exploring and quantifying the potential direct benefits of GLP-1 RAs can provide a more comprehensive understanding of their overall impact on patient health outcomes. Furthermore, this study specifically focused on patients inadequately controlled with basal insulin and did not include other populations. Therefore, the generalizability of our results to other groups, such as those with inadequate control of oral hypoglycemic agents, may be restricted. Lastly, some input parameters, such as health utility, were not China-specific due to a lack of data. Reassuringly, the results of the sensitivity analysis showed minimal impact on study results.

## 5 Conclusion

From the perspective of Chinese healthcare payers, our findings suggest that once-daily oral semaglutide as an add-on therapy is likely to be a more cost-effective option than most injectable GLP-1 RAs in China, excluding injectable semaglutide and exenatide, for patients with T2DM who have inadequate control on basal insulin. To achieve cost-effectiveness compared to placebo, a moderate reduction in the price of oral semaglutide below $1,711.03 per year may be necessary in the market. Our study not only highlights the potential benefits of GLP-1 RAs in enhancing the health-related quality of life but also provides valuable insights into the cost-effectiveness of oral semaglutide in poorly controlled Chinese patients with T2DM on basal insulin. These insights have significant implications for both payers and clinicians in their decision-making processes. However, to validate these findings, further research, including cost-effectiveness models and head-to-head clinical trials in Chinese patients, is warranted.

## Data Availability

The raw data supporting the conclusion of this article will be made available by the authors, without undue reservation.
