# Supplementary material for: Cost-effectiveness analysis of once-daily oral semaglutide versus placebo and subcutaneous glucagon-like peptide-1 receptor agonists added to insulin in patients with type 2 diabetes in China
Source: Front Pharmacol. 2023 Aug 9;14:1226778. doi: 10.3389/fphar.2023.1226778 (PMC10445164; doi:10.3389/fphar.2023.1226778)
Supplement: Supplementary file 1 [file DataSheet1.docx]

Supplementary Material

Title Cost-effectiveness analysis of once-daily oral semaglutide versus placebo and subcutaneous glucagon-like peptide-1 receptor agonists added to insulin in patients with type 2 diabetes in China

Zhen Feng, Wai Kei Tong, Xinyue Zhang, Zhijia Tang^*^

*** Correspondence:** Zhijia Tang [zjtang@fudan.edu.cn](mailto:zjtang@fudan.edu.cn)

# Supplementary Tables

Table S1. Baseline characteristics and risk factors of the simulation cohort.

| Characteristics | Mean | SD (%) |
| --- | --- | --- |
| Total simulation sample | 1000 (each group) |  |
| Race, % |  |  |
| White | 57.4 |  |
| Black/African American | 6.1 |  |
| Asian-Indian | 36.5 |  |
| Gender, % |  |  |
| Female | 53 |  |
| Mean age, years | 61.0 | 10.0 |
| Duration of diabetes, years | 14.1 | 8.0 |
| BMI, kg/m^2^ | 30.8 | 6.3 |
| Mean body weight, kg | 84.6 | 21.0 |
| AF | 1% |  |
| PVD | 13% |  |
| Smoker | 21% |  |
| Albuminuria | 19% |  |
| HDL | 1.2 |  |
| LDL | 2.36 |  |
| SBP | 133 |  |
| Mean HbA1c, % | 8.2 | 0.7 |
| Heart rate, bmp | 74 |  |
| WBC | 6.9 | 2.2 |
| Hemoglobin | 14.5 | 1.6 |
| eGFR, ml/min/1.73 m^2^ | 91 | 14 |

Abbreviations: BMI: body mass index; AF: atrial fibrillation; PVD: Peripheral vascular disease; HDL: high-density lipoprotein cholesterol; LDL: low-density lipoprotein cholesterol; SBP: systolic blood pressure; HbA1c: hemoglobin A1c; WBC: White blood cell; eGFR: estimated glomerular filtration rate.

Table S2. Estimated treatment difference between once-daily oral semaglutide 14 mg and comparators in network meta-analysis.

| Comparators | Mean treatment difference (95% CI) | | |
| --- | --- | --- | --- |
|  | HbA1c, % | Body weight, kg | SBP, mmHg |
| Placebo | -1.62 (-1.93, -1.31) | -3.71 (-4.73, -2.67) | -2.66 (-6.57, 1.27) |
| Sc. semaglutide 1.0 mg QW | 0.08 (-0.32, 0.47) | 1.30 (-0.17, 2.76) | 3.66 (-1.57, 8.98) |
| Dulaglutide 1.5 mg QW | -0.85 (-1.25, -0.45) | -1.28 (-2.61, 0.03) | NA |
| Liraglutide 1.8 mg OD | -0.42 (-0.78, -0.05) | -0.61 (-1.87, 0.66) | 2.36 (-2.14, 7.02) |
| Exenatide10 ug BID | -0.93 (-1.32, -0.54) | -0.96 (-2.41, -0.48) | 1.74 (-3.39, 6.87) |
| Lixisenatide 20 ug OD | -1.32 (-1.74, -0.91) | -2.39 (-3.66, -1.14) | NA |

Treatment difference: once-daily oral semaglutide vs comparators. Abbreviations: BID twice-daily; CI confidential interval; NA not available; OD once-daily; QW once-daily; SBP systolic blood pressure; Sc. subcutaneous.

Table S3. Costs of medications.

| Medication | Unit of cost (CNY/box) | Specification | Usual dosage | ^a^Annual cost (CNY) | ^b^Annual cost (USD) | ^c^Insurance year |
| --- | --- | --- | --- | --- | --- | --- |
| Sc. SEMA | 478.80 | 1.34 mg/ml, 1.5 ml, one piece/box | 1 mg QW | 12,448.8 | 1,871.9 | 2021 |
| Oral SEMA | NA | 14 mg | 14 mg OD | 12,448.8 | 1,871.9 | NA |
| DULA | 298 | 3 mg | 1.5 mg QW | 7,748 | 1,165.0 | 2017 |
| Liraglutide | 315.27 | 3 ml: 18 mg | 1.8 mg OD | 11,475.8 | 1,725.6 | 2020 |
| Exenatide | 408 | 10 ug (0.25 mg/ml, 2.4 ml/piece) | 10 ug BID | 4,950.4 | 744.4 | 2019 |
| Lixisenatide | 268 | 20 ug (0.10 mg/ml, 3 ml/piece) | 20 ug OD | 6,503.5 | 977.9 | 2019 |

^a^ 52 week per year; ^b^ 1 USD = 6.6504 CNY; ^c^ time to enter the national medical insurance catalogue. Abbreviations: Sc. SEMA: subcutaneous semaglutide; DULA: dulaglutide; LIRA: liraglutide; EXEN: exenatide; LIXI: lixisenatide. BID twice-daily; CNY: Chinese Yuan; NA not available; OD once-daily; QW once-daily; USD: United States dollar.

Table S4. Key model inputs of costs for diabetes-related complications and health state utility decrements.

| Model input | At time of event | | | In subsequent years | |  |
| --- | --- | --- | --- | --- | --- | --- |
|  | Fatal cost (1) | Non-fatal cost (1) | Utility decrement | Cost | Utility decrement | 95% CI |
| IHD | 0 | 6751.19 | -0.09 (2, 3) | 1205.25 | -0.09 (2, 3) | -0.126~-0.054 |
| MI | 8426.67 | 8426.67 | -0.055 (2, 3) | 519.78 | -0.236 (4) | 0.026~0.446 |
| Heart failure | 3254.46 | 3254.46 | -0.236 (4) | 1720.83 | -0.236 (4) | 0.026~0.446 |
| Stroke | 2431.21 | 3281.64 | -0.164 (2, 3) | 578.56 | -0.326 (4) | 0.036~0.616 |
| Amputation | 4757.25 | 4757.25 | -0.380 (2, 5-9) | 4630.73 | -0.380 (2, 5-9) | 0.264~0.496 |
| Blindness | / | 2533.31 | -0.157 (2, 5-9) | 1874.11 | -0.157 (2, 5-9) | 0.007~0.307 |
| Renal failure | 0 | 15754.43 | -0.400 (2, 5-9) | 15754.43 | -0.400 (2, 5-9) | 0.190~0.610 |
| Ulcer | / | 2478.07 | -0.059 (1, 2) | 872.16 | -0.059 (1, 2) |  |
| Initial utility | 0.876 (4) |  |  |  |  | 0.780~0.920 (1) |
| Cost in diabetes management | 1530.93 |  |  |  |  |  |

Note: Costs were collected from published literatures on Chinese economic evaluation and expressed in 2022 US dollars. Abbreviations: IHD: ischemic heart disease; MI: myocardial infarction.

Table S5. Input parameters for univariate sensitivity analysis.

| Parameters | Lower boundary value | Upper boundary value | Range source |
| --- | --- | --- | --- |
| Model settings |  |  |  |
| Discount rate, % | 3 | 8 | (1) |
| Time horizon | 30 | 50 | (1) |
| Treatment time, years | 4 | 6 | (1) |
| Absolute treatment effects |  |  |  |
| HbA1c treatment effect, % | -2.23 | -1.5 | (10) |
| Weight treatment effect, % | -5.08 | -2.67 | (10) |
| Systolic blood pressure treatment, mmHg | -7.51 | 0.71 | (10) |
| Costs |  |  |  |
| Annual oral semaglutide cost | 1497.52 | 2246.28 | ±20% |
| Diabetes management cost | 1224.74 | 1837.12 | ±20% |
| IHD per year cost | 964.20 | 1446.30 | ±20% |
| MI per year cost | 415.82 | 623.74 | ±20% |
| HF per year cost | 1376.66 | 2065.00 | ±20% |
| Stroke per year cost | 462.85 | 694.27 | ±20% |
| Amputation per year cost | 3704.58 | 5556.88 | ±20% |
| Blindness per year cost | 1499.29 | 2248.93 | ±20% |
| Renal failure per year cost | 12603.54 | 18905.32 | ±20% |
| Ulcer per year cost | 697.73 | 1046.59 | ±20% |
| Health utility |  |  |  |
| Initial utility | 0.780 | 0.920 | (1) |
| IHD disutility scores | 0.054 | 0.126 | 95% CI |
| MI disutility scores | 0.026 | 0.446 | 95% CI |
| HF disutility scores | 0.026 | 0.446 | 95% CI |
| Stroke disutility scores | 0.036 | 0.616 | 95% CI |
| Amputation disutility scores | 0.264 | 0.496 | 95% CI |
| Blindness disutility scores | 0.007 | 0.307 | 95% CI |
| ERSD disutility scores | 0.190 | 0.610 | 95% CI |
| Ulcer disutility scores | 0.047 | 0.071 | ±20% |

# Note: Range of costs and disutility scores were collected from published literatures on Chinese economic evaluation. Unreported values were tested as ±20%.

# Reference

1. Hu S, Gu S, Qi C, Wang S, Qian F, Shi C, et al. Cost-utility analysis of semaglutide for type 2 diabetes after its addition to the National Medical Insurance System in China. Diabetes Obes Metab. 2022.<https://doi.org/10.1111/dom.14881>

2. Gu S, Mu Y, Zhai S, Zeng Y, Zhen X, Dong H. Cost-Effectiveness of Dapagliflozin versus Acarbose as a Monotherapy in Type 2 Diabetes in China. PLoS One. 2016;11(11):e0165629.<https://doi.org/10.1371/journal.pone.0165629>

3. Clarke P, Gray A, Holman R. Estimating utility values for health states of type 2 diabetic patients using the EQ-5D (UKPDS 62). Med Decis Making. 2002;22(4):340-9.<https://doi.org/10.1177/0272989X0202200412>

4. Pan CW, Sun HP, Zhou HJ, Ma Q, Xu Y, Luo N, et al. Valuing Health-Related Quality of Life in Type 2 Diabetes Patients in China. Med Decis Making. 2016;36(2):234-41.<https://doi.org/10.1177/0272989X15606903>

5. Hou X, Wan X, Wu B. Cost-Effectiveness of Canagliflozin Versus Dapagliflozin Added to Metformin in Patients With Type 2 Diabetes in China. Front Pharmacol. 2019;10:480.<https://doi.org/10.3389/fphar.2019.00480>

6. Li T, Liu M, Ben H, Xu Z, Zhong H, Wu B. Clopidogrel versus aspirin in patients with recent ischemic stroke and established peripheral artery disease: an economic evaluation in a Chinese setting. Clin Drug Investig. 2015;35(6):365-74.<https://doi.org/10.1007/s40261-015-0290-x>

7. Wang H, Lin X, Zhang Z, Wang Q, Chen JM, Liu J, et al. The economic burden of inpatients with type 2 diabetes: a case study in a Chinese hospital. Asia Pac J Public Health. 2015;27(2 Suppl):49S-54S.<https://doi.org/10.1177/1010539515572220>

8. Shao H, Zhai S, Zou D, Mir MU, Zawadzki NK, Shi Q, et al. Cost-effectiveness analysis of dapagliflozin versus glimepiride as monotherapy in a Chinese population with type 2 diabetes mellitus. Curr Med Res Opin. 2017;33(2):359-69.<https://doi.org/10.1080/03007995.2016.1257978>

9. Wu B, Wan X, Ma J. Cost-effectiveness of prevention and management of diabetic foot ulcer and amputation in a health resource-limited setting. J Diabetes. 2018;10(4):320-7.<https://doi.org/10.1111/1753-0407.12612>

10. Chubb B, Gupta P, Gupta J, Nuhoho S, Kallenbach K, Orme M. Once-Daily Oral Semaglutide Versus Injectable GLP-1 RAs in People with Type 2 Diabetes Inadequately Controlled on Basal Insulin: Systematic Review and Network Meta-analysis. Diabetes Ther. 2021;12(5):1325-39.<https://doi.org/10.1007/s13300-021-01034-w>
